# OTEH-9. scRNA sequencing of proneural GBM avatar model reveals acquisition of oncogenic transcriptional programming and infers a developmental path towards a genomically unstable state

**DOI:** 10.1093/noajnl/vdab070.048

**Published:** 2021-07-05

**Authors:** Brett M Taylor, Isaac A Chaim, Tomoyuki Koga, Shunichiro Miki, Brian E Yee, Gene W Yeo, Frank B Furnari

**Affiliations:** 1 Ludwig Cancer Research San Diego Branch, La Jolla, CA, USA; 2 Department of Pathology, University of California San Diego, La Jolla, CA, USA; 3 Department of Cellular and Molecular Medicine, University of California San Diego, La Jolla, CA, USA; 4 Institute for Genomic Medicine, University of California San Diego, La Jolla, CA, USA; 5 Department of Neurosurgery, University of Minnesota, Minneapolis, MN, USA

## Abstract

Glioblastomas (GBMs) are the most common malignant primary brain tumors, and the paucity of novel treatments warrants an investigation of its origins and development into aggressive, lethal tumors. Koga & Chaim *et al.* have recently shown that human pluripotent stem cells (hiPSCs) with different combinations of driver mutations can be differentiated into neural progenitor cells (NPCs) and engrafted into mice to form high grade gliomas (iHGGs). In this work, scRNA seq analysis was used to investigate the development of *TP53*^*−/−*^*;PDGFRA*^*Δ8–9*^ iHGGs, an avatar model that has been shown to recapitulate the proneural subtype of GBM. After re-engrafting the primary avatar cultures (secondary tumor stage), the *TP53*^*−/−*^*;PDGFRA*^*Δ8–9*^ iHGGs developed diverse transcriptional programming and acquired a subpopulation of cells with high expression of known GBM oncogenes, such as MYC, CDK4, and PDGFRA. Notably, when all datasets were aggregated, this oncogene amplifying transcriptional program became the largest source of variation between all stages and replicates of the *TP53*^*−/−*^*;PDGFRA*^*Δ8–9*^ iHGGs. Indicated by a larger total copy number variation (CNV), this oncogene-amplifying program was associated with a genomically unstable developmental state. Trajectory inference could track the development of this population from the initial primary culture of *TP53*^*−/−*^*;PDGFRA*^*Δ8–9*^ iHGG. Differential gene expression analysis identified distinct divergences in clonal evolution––e.g., high expression of the S100 protein family in one cluster––following the acquisition of this genomically unstable state. Lastly, genomic PCR was used to ascertain whether these changes in transcriptional programming were reflected in changes in DNA copy number and identified DNA amplifications of MYC and CDK4. Our scRNA seq analysis of the GBM avatar model platform provides novel insight into how oncogenic states in GBM develop from a small number of driver mutations.

